# Access to Chemical Data: Lutter et al. Respond

**DOI:** 10.1289/ehp.1206438R

**Published:** 2013-04-01

**Authors:** Randall Lutter, Craig Barrow, Christopher J. Borgert, James W. Conrad, Debra Edwards, Allan Felsot

**Affiliations:** 1 Independent Consultant, Bethesda, Maryland, E-mail: rwlutter@gmail.com; 2 Craig Barrow Consulting, Gibsonia, Pennsylvania; 3 Applied Pharmacology and Toxicology Inc., Gainesville, Florida; 4 Conrad Law & Policy Counsel, Washington, DC; 5 Independent Consultant, Alexandria, Virginia; 6 Food and Environmental Quality Lab, Washington State University, Richland, Washington

We appreciate the attention paid by Goldman and Silbergeld (2013) to the issue of data disclosure and agree that there has been “increased demand for transparency and disclosure of the data used by the U.S. EPA [Environmental Protection Agency] to make evaluations that support regulatory decisions.”

In their letter, Goldman and Silbergeld contend primarily that “replication” in science means to independently repeat a prior study to see if the same results can be obtained. They suggest that public availability of the prior study’s data is unnecessary because a subsequent study will generate its own data. In 2011, a special section of *Science* (Vol. 334, No. 6060) addressed replicability and reproducibility and made two general points. First, “replication,” as defined by Goldman and Silbergeld, while perhaps the cornerstone of the scientific method, can be difficult in many settings because of the uniqueness of the precise conditions surrounding field observations, the expense and time required to collect data (e.g., for longitudinal studies), and ethical constraints (e.g., Jasny et al. 2011). Second, in those cases where conduct of a second experiment may be impossible or infeasible, review and reanalysis of the first study’s data is still a meaningful step along the “reproducibility spectrum,” assists in understanding the differences between competing analyses, and “may be sufficient to verify the quality of the scientific claims” (Peng 2011; see also Ioannides and Khoury 2011; Santer et al. 2011).

Other empirical work also supports the view that data availability promotes reproducibility. In empirical economics, a discipline that uses large-scale statistical models broadly similar to those of epidemiologists, a famous study of replication of peer-reviewed research suggested that inadvertent errors may be “commonplace rather than rare occurrences” (Dewald et al. 1986). The *American Economic Review* (AER 2013) subsequently adopted a policy “to publish papers only if the data used in the analysis are clearly and precisely documented and are readily available to any researcher for purposes of replication.” Further, the *AER* conducted a recent evaluation of its policy and reported that about 80% of 39 sampled papers met the spirit of the data availability policy (Glandon 2010). Importantly, independent efforts at replication of 9 selected papers found no serious errors (almost exact replication for 5 studies and “several small discrepancies … immaterial to the conclusions” for another 4.) This result represents a marked improvement relative to the results of the original 1986 study of replication. The difference is presumably attributable, at least in part, to the difference in care and quality of work associated with the *AER*’s current policy of data availability. Although analytic methods underlying papers published in the *AER* are different from those used in chemical evaluation, the experience of the *AER* suggests that there is merit in promoting data availability for the purpose of improving the reliability of the results of published, peer-reviewed scientific papers, at least in disciplines that use complex statistical models.

Finally, we, like Goldman and Silbergeld, “disagree with the argument that raw data from every study used by the U.S. EPA to support a regulatory assessment should be made available to the agency and to the public.” Unlike Goldman and Silbergeld, we recom-mend that the U.S. EPA, when it uses results of a published study in a regulatory assessment, ask the authors for under-lying data (Lutter et al. 2013). If the U.S. EPA does not receive such data, it should explain how it used the study results in light of the fact that data sufficient to assess reproducibility was not forthcoming. We believe our approach would facilitate and not obstruct good science and that it would not discourage researchers from studying issues of importance in environ-mental health. Moreover, it would not, as Goldman and Silbergeld state,

limit the U.S. EPA from using the results of research published in the peer-reviewed scientific literature by placing studies off-limits if the authors did not submit raw data sets to the U.S. EPA.
